# Effect of the Topology and Delayed Interactions in Neuronal Networks Synchronization

**DOI:** 10.1371/journal.pone.0019900

**Published:** 2011-05-27

**Authors:** Toni Pérez, Guadalupe C. Garcia, Víctor M. Eguíluz, Raúl Vicente, Gordon Pipa, Claudio Mirasso

**Affiliations:** 1 Department of Physics, Lehigh University, Bethlehem, Pennsylvania, United States of America; 2 School of Engineering and Science, Jacobs University Bremen, Bremen, Germany; 3 Instituto de Física Interdisciplinar y Sistemas Complejos (CSIC-UIB), Palma de Mallorca, Spain; 4 Department of Neurophysiology, Max Planck Institute for Brain Research, Frankfurt, Germany; 5 Frankfurt Institute for Advanced Studies, Frankfurt, Germany; 6 Institute of Cognitive Science, University of Osnabrueck, Osnabrueck, Germany; University of Maribor, Slovenia

## Abstract

As important as the intrinsic properties of an individual nervous cell stands the network of neurons in which it is embedded and by virtue of which it acquires great part of its responsiveness and functionality. In this study we have explored how the topological properties and conduction delays of several classes of neural networks affect the capacity of their constituent cells to establish well-defined temporal relations among firing of their action potentials. This ability of a population of neurons to produce and maintain a millisecond-precise coordinated firing (either evoked by external stimuli or internally generated) is central to neural codes exploiting precise spike timing for the representation and communication of information. Our results, based on extensive simulations of conductance-based type of neurons in an oscillatory regime, indicate that only certain topologies of networks allow for a coordinated firing at a local and long-range scale simultaneously. Besides network architecture, axonal conduction delays are also observed to be another important factor in the generation of coherent spiking. We report that such communication latencies not only set the phase difference between the oscillatory activity of remote neural populations but determine whether the interconnected cells can set in any coherent firing at all. In this context, we have also investigated how the balance between the network synchronizing effects and the dispersive drift caused by inhomogeneities in natural firing frequencies across neurons is resolved. Finally, we show that the observed roles of conduction delays and frequency dispersion are not particular to canonical networks but experimentally measured anatomical networks such as the macaque cortical network can display the same type of behavior.

## Introduction

A self-organized coordination between individual agents is often the hallmark of many natural and man-made complex systems. One of the most prominent examples of such behavior is the phenomenon of synchronization [1], [2]. Synchronization arises in systems of different origin such as mechanical oscillators, lasers, chemical reactions, cell populations, or social interactions [3]. For instance, there is experimental evidence for rhythmic and correlated firing of neurons, although the functional role of such collective dynamics is still at debate [4]. To elucidate this question it is necessary to describe the parameter space and the mechanisms underlying the variety of neuronal oscillations and synchrony that has been reported [5].

The exact conditions under which large populations of neurons spontaneously synchronize are in general not fully understood, even in the non-delayed coupling case. In the latter case, Mirollo and Strogatz analytically demonstrated that synchrony can be a stable state for a population of globally pulse-coupled oscillators [6]. However, to do the analytics feasible, most of these works considered systems composed of few identical neurons (typically two), or homogeneous topologies such as the all-to-all network. Numerical studies have incorporated the features of non-homogeneities and complex network structures into the analysis of neuronal populations. For instance, the detailed role of the nodes degree distribution, long range connections, average path length, and clustering on the level of synchronization and oscillatory behavior of the network have been addressed [7]–[9].

Delay in the interaction among dynamical systems has an ambivanlent effect. In some cases it can stabilize the systems [10], increase synchronization [11], induce stochastic resonance [12] or enhance coherence of spiral waves [13]. In other cases, it can completely destabilize the system yielding chaotic dynamics [14]. In large networks of neurons, for instance, it has been observed a wide variety of spatio-temporal patterns and the existence of various regions of multistability [15]. Delays arising from the propagation of action potential in neuronal systems can amount to several tens of milliseconds [16], [17]. These latencies add an intrinsic component to the timing of individual spikes which might have important consequences at the network level and whose understanding is still to be clarified [18]. Investigations taking into account delays remain scarce [19]. Globally delay-coupled maps show that inhibitory coupling enhance in-phase synchronization while excitatory coupling leads to out-of-phase synchronization [20]–[22]. In small world networks, short or moderate conduction delays favor synchronization for both chemical and electrical coupling, while long conduction delays always evoke antiphase synchronization and clustering [23].

Delay-induced sychronization in complex networks of bursting nodes modeled with Rulkov maps has been recently studied. While an increase of the coupling strength always enhance synchronization, regular or irregular propagating fronts appear intermitently as delays increase [13], [24]. Interestingly, depending on the coupling mechanism, either attractive or repulsive, minima (maxima) of the synchronization quantifiers are oberved at the delay time and multiples of it for attractive (repulsive) coupling [25]. These results are opposite to what we find for the case of spiking neurons as will be discussed later.

It is also reported that diffusive delayed coupling enhance synchrony [26] and leads to phase clusterization [27]. Some studies have specifically focused on the long-distance synchronization and proposed some canonical circuits that naturally promote zero-lag synchrony [11], [28].

## Materials and Methods

**Figure 1:**
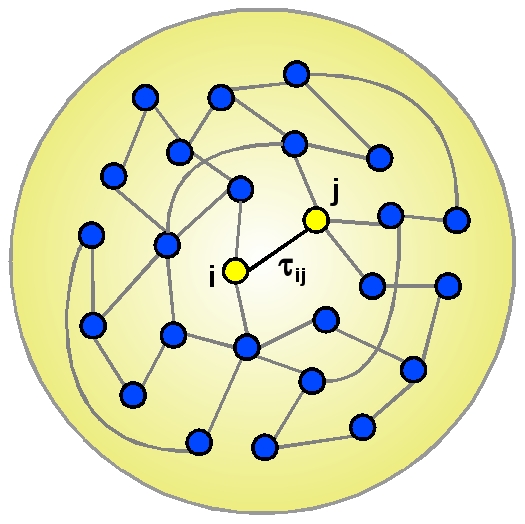
Sketch of the system under study. Schematic representation of the neuronal network with delay interactions.

Our aim is to study the interplay between conduction delays and network topology in an ensemble of delay interconnected neurons. First, we address the ideal situation where all conductance delays are identical and later the situation where the natural frequencies of the neurons are distributed. In order to understand the role played by the pathways in which the neurons interact with other neurons, we consider different interconnection topologies, ranging from regular one-dimensional lattices to scale-free networks. Figure 1 shows a schematic representation of the system.

### Description of neuron dynamics

We study two types of neural excitability by mean of two different conductance-based models. In these models, the membrane potential 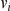 of the neuron 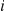 is described as
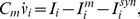
(1)where 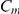 is the membrane capacitance per unit area; 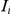 is the external current; 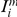 is the membrane current and 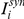 is the synaptic current.

In the Hodgkin and Huxley (HH) model [29], the membrane current is described by
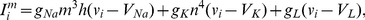
(2)where 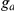 (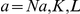) represents the maximum conductance for the ionic contributions and passive channel respectively and 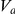 are the corresponding equilibrium potentials. The gating variables 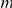, 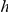, and 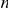 represent the activation and inactivation of the sodium channels and the activation of the potassium channels, respectively. These voltage-gated ion channels are described by the following differential equation
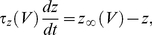
(3)where 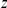 denotes a generic gating variable. The functions 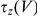 and 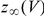 are determined from experimental data, and take the form
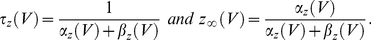
(4)


In the Connor-Stevens (CS) model [30], the equation describing the membrane current reads:
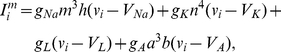
(5)


This model includes an additional conductance current, called the A-current, which is responsible of the different excitatory behaviors between both models [31]. The experimentally fitted voltage-dependent transition rates 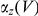 and 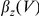 and the rest of parameters values for these models can be found in Text S1, Table S1 and Table S2.

In both models, the pulsed synaptic transmission between neurons is modeled, following [32], by a postsynaptic conductance change with the form of an alpha-function. The synaptic current is defined as
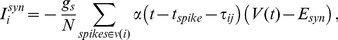
(6)where 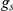 describes the maximal synaptic conductance and the sum is extended over the train of presynaptic spikes occurring at 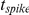 produced by neighbors of the neuron 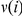. The reversal potential 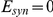 mV defines the synaptic connection as excitatory. The alpha-function takes the form
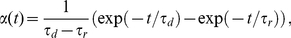
(7)where the parameters 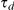 and 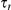 stand for the decay and rise time of the function and determine the duration of the response. Synaptic rise and decay times were set to 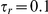 and 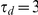 ms, respectively. The delay arising from the finite conduction velocity of axons is taken into account through the latency time 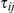. In this work we consider the situation in which all the conduction delays were set to the same value 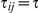.

Neurons can also interact with each other through electrical synapses, also known as gap junctions. In the electrical synaptic case, the synaptic current takes the form:
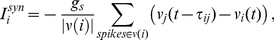
(8)


We study how both synaptic transmission schemes affect the network synchronization.

### Interconnection topology

The synchronization of neurons may depend on the synaptic network in which they are embedded. To study the role played by the different synaptic pathways in the synchronization of our ensemble of neurons, we consider five different topologies such as: regular, small-world, random, scale-free and globally coupled networks. In the regular lattice, neurons are connected with the 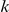 nearest-neighbors using periodic boundary conditions. To construct a small-world network we use the algorithm proposed by Watts and Strogatz [33]. The algorithm starts from a regular lattice and with a certain probability 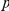 each link is rewired to another node randomly chosen from all possible nodes that avoid self-loops and link duplications. In the limit in which the rewiring probability is one, we obtain the random network. The scale-free network was introduced by Barabási and Albert [34] and is based on a preferential attachment mechanism. The main feature of this network is that the degree distribution follows a power-law distribution. Most of the nodes are connected with few elements and only a few nodes are connected with many elements. For all these topologies the average degree is 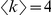. We will also consider the all-to-all network where neurons are connected with all others neurons.

### Phase Synchronization

To characterize the synchronization in our network, we define the phase [35] of neuron 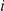 as:
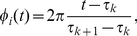
(9)where 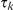 is the time of the *k*th firing of the neuron *i*. The idea behind this definition is that the phase of a neuron experiments a change of 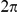 between two consecutive spikes. To measure the phase synchronization between neuron 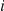 and the set of its neighbors 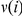, we define the quantity:
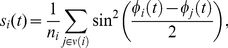
(10)with 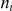 the degree of neuron 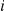, i.e., the number of connected neighbors of the neuron *i*. Averaging over elements and integrating in time, we obtain
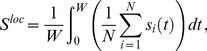
(11)where 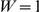 s is the length of the time window used for averaging and 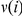 is the set of neighbors of the neuron 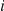. 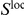 gives a measure of the average of the local phase synchronization in the coupled system.

To measure global synchronization we extend the sum in Eq. (10) to all neurons. Then, we quantify the global phase synchronization of neuron 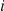 with the rest of the network as
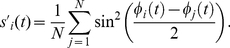
(12)


Averaging over elements and time we obtain a global order parameter.
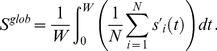
(13)


These order parameters are zero if the phases of the neurons are equal and one if they differ by 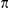. When the phases of the neurons are randomly distributed, the order parameters take a value of 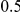.

## Results

First we analyze the case of identical neurons. We study the effect that the rewiring probability and the number of connected neighbors have on the synchronization of the system. Then, we consider the situation in which the natural frequencies of the neurons are distributed according to a Gaussian distribution.

Finally, we particularize our study to a real network, the macaque cortico-cortical network.

### Homogeneous frequencies

Let us consider the situation in which the delays in the connection between neurons are all identical and the neurons operate in a regular spiking regime (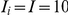 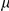
A cm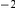). In this situation, all neurons fire with the same natural frequency 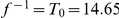 ms when isolated.

**Figure 2:**
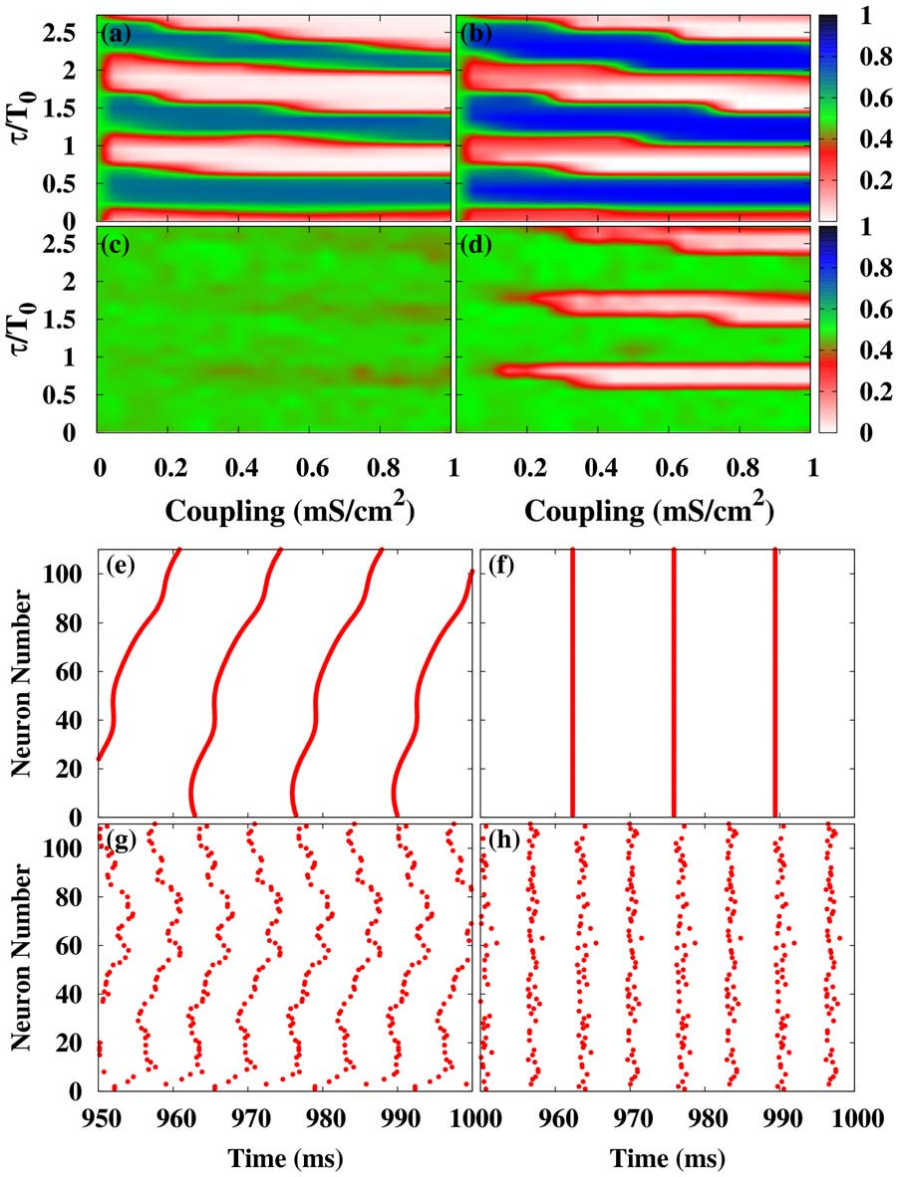
Local and global synchronization regions and rater plots for different networks. (a-d) Contour plots of 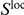 [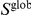] for the HH neuron model in the coupling strength-delay time phase space corresponding to (a) [(c)] a SWN (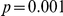) and (b) [(d)] a RN. In panels (e-h) we show raster plots of a fraction of the neurons for different networks and delays: 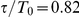 [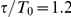] in (e) [(g)] for a SWN (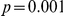) and (f) [(h)] for a RN. The coupling strength is 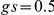 mS cm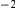.

In Fig. 2(a–d) we show, for the HH neuronal model, the contour plots of 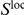 and 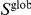 in the coupling strength vs. normalized delay for a small-world network (SWN) and a random network (RN). At a local level, three different regimes are observed: (i) *In*-phase solutions (white regions in Fig. 2(a–b)) appear for delays close to multiples of the natural period 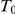 of the neurons. In this regime, neighboring neurons fire with almost the same phase. (ii) *Anti*-phase solutions (blue regions in Fig. 2(a–b)) emerge for delays close to odd multiples of the half of the natural period. In this case, neighboring neurons fire with a phase difference of 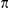 between them. (iii) *Out-of*-phase solutions (green regions in Fig. 2(a–b)) arise between the two previous regimes. In this regime, the neurons fire with a random phase difference between them.

At a global scale however, in-phase synchronization is observed in the RN while it is absent in the SWN (see Fig. 2(c–d)). The time traces of the neurons give us more insight of those dynamical regimes. Figure 2(e–h) shows raster plots of a fraction of neurons in a SWN and a RN, for a fixed coupling strength and two different values of 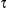. Although at a local scale the synchronized regimes, either in- or anti-phase, are observed in different networks, the nature of the synchronized state is, however, different depending on the underlying topology. In the SWN (and in the one-dimensional regular lattice) local synchronization is attained in fronts that propagate through the network. This synchronization, observed at a local scale for the SWN, disappears at a global scale when averaging over the entire network as shown in Fig. 2(c). In the RN, however, perfect in-phase synchronization is observed for some values of the delay yielding an in-phase state at a global scale, as shown in Fig. 2(d). For the anti-phase state, the raster plot reveals pulsations with a phase difference of 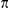 between neighboring neurons but with a significant jitter. This local anti-phase firing leads to a 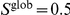 and consequently an apparent absence of global anti-phase synchronization in Fig. 2(d). Similar scenario is observed in the scale-free network.

**Figure 3:**
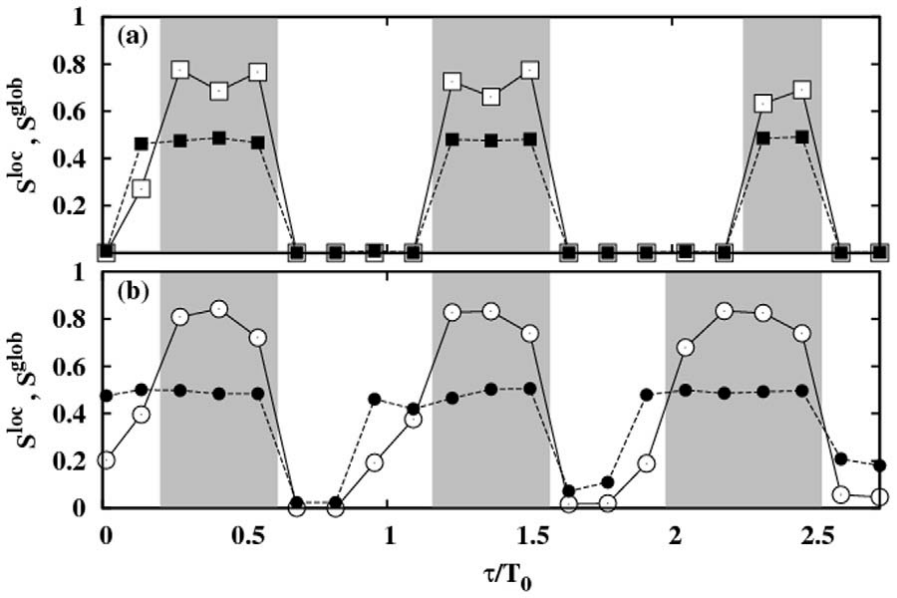
Section of the local and global synchronization regions for different coupling schemes. 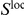
 (open symbols) and 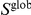 (solid symbols) as a function of the delay 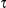 in a RN when the neurons interact via (a) electrical coupling and (b) chemical coupling. Gray areas represent the regions where anti-phase synchronization is observed. Coupling strength is fixed to 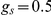 mS cm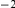.

To explore the generality of the previous synchronization regions, we also analyzed the case of electrical coupling. In Fig. 3 we compare 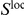 and 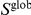 in a RN for a fixed value of the coupling strength in both electrical and chemical couplings. The synchronization regions remain very similar in both cases, although the electrical coupling favors the in-phase synchronization increasing the areas where it appears. However, the alternation between in-phase and anti-phase regions remains qualitatively similar in both cases (see grey areas in Fig. 3). This tendency to in-phase synchronization is more dramatic in the all-to-all network, where the resonant role of the delay is even lost.

**Figure 4:**
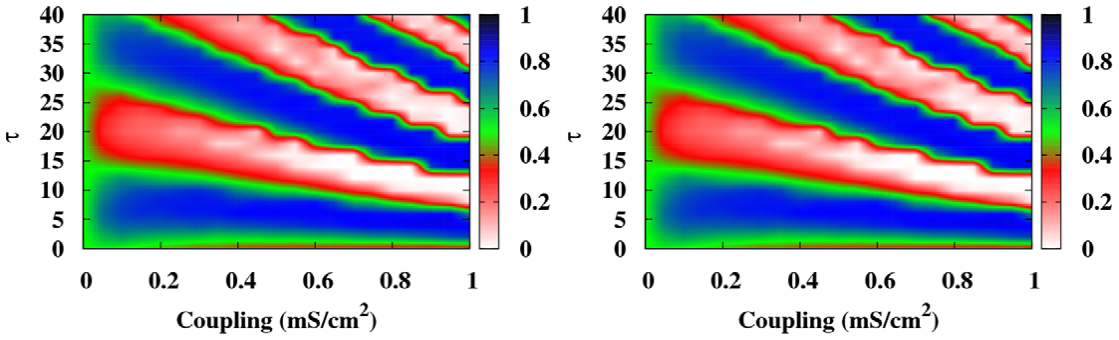
Local and global synchronization regions for the CS dynamics. 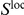
 (left) and 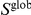 (right) for the chemically (pulsed) delay-coupled CS neuronal model in a random network.

We have also checked that different neuron dynamics like that described by the Connor-Stevens (CS) model exhibits qualitatively the same synchronization regions. In Fig. 4 we show the contour plots of 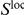 and 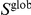 in the coupling strength vs. normalized delay for a random network for the CS neuronal model. Pulsed coupling yields the same qualitatively result except for the firing frequency dependence on the coupling strength.

### Effect of the rewiring and coupling

**Figure 5:**
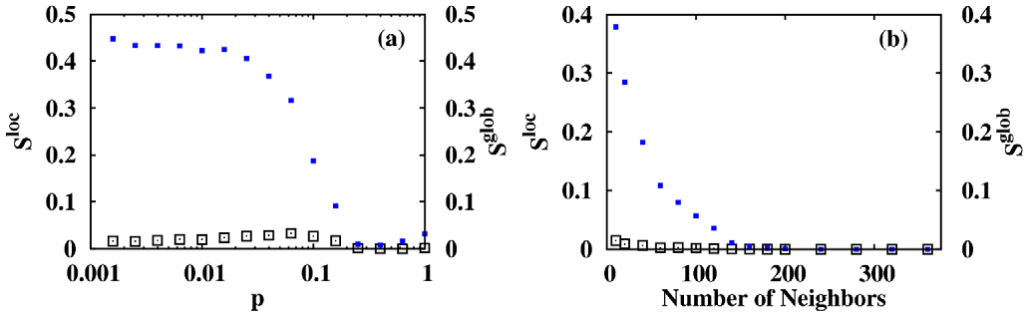
Evolution of the local and global synchronization indexes with different network properties. Dependence of 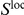 (open squares) and 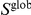 (solid squares) on (a) the rewiring and (b) the number of neighbors in the network. Other parameter values are: coupling strength 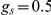 mS cm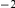 and delay time 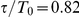.

In order to understand the role played by large-range interconnections, we randomize the regular one-dimensional lattice. Figure 5(a) shows, for the HH model, the global and local order parameter as a function of the rewiring probability in the network. Initially, for a delay 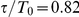 and a coupling 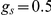 mS cm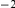, the activity of the network is only locally synchronized. Increasing the rewiring probability, the activity of the network becomes globally synchronized. We also investigate in the regular lattice, the effect of increase the number of neighbors (Fig. 5(b)). We observe a gradual transition from a global desynchronized state to a synchronized one for a fraction of neighbors around 10%.

**Figure 6:**
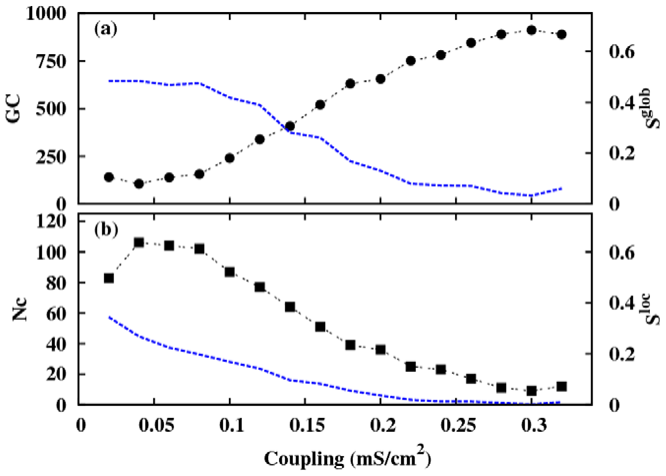
Evolution of the giant component and the number of clusters with the interaction strength. Dependence on the coupling strength of (a) giant component 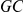 (circles) and 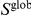 (dashed line) and, (b) number of clusters 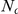 (squares) and 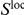 (dashed line) in a RN. The delay time is 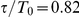.

With the aim of investigating the mechanism towards the synchronization in our system, we show in Fig. 6 the number of clusters (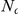) of synchronized neurons and the size of the giant component (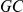) together with 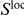 and 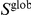, as a function of the coupling strength [36]. Although the system is not initially synchronized neither locally nor globally, small clusters of synchronized neurons emerge. As the coupling increases and the system moves towards a synchronized state, these small clusters gradually merge developing the largest cluster, that, eventually, reaches the network size, which suggests a similar behavior as the one observed in the absence of delay [36].

### Heterogeneous frequencies

**Figure 7:**
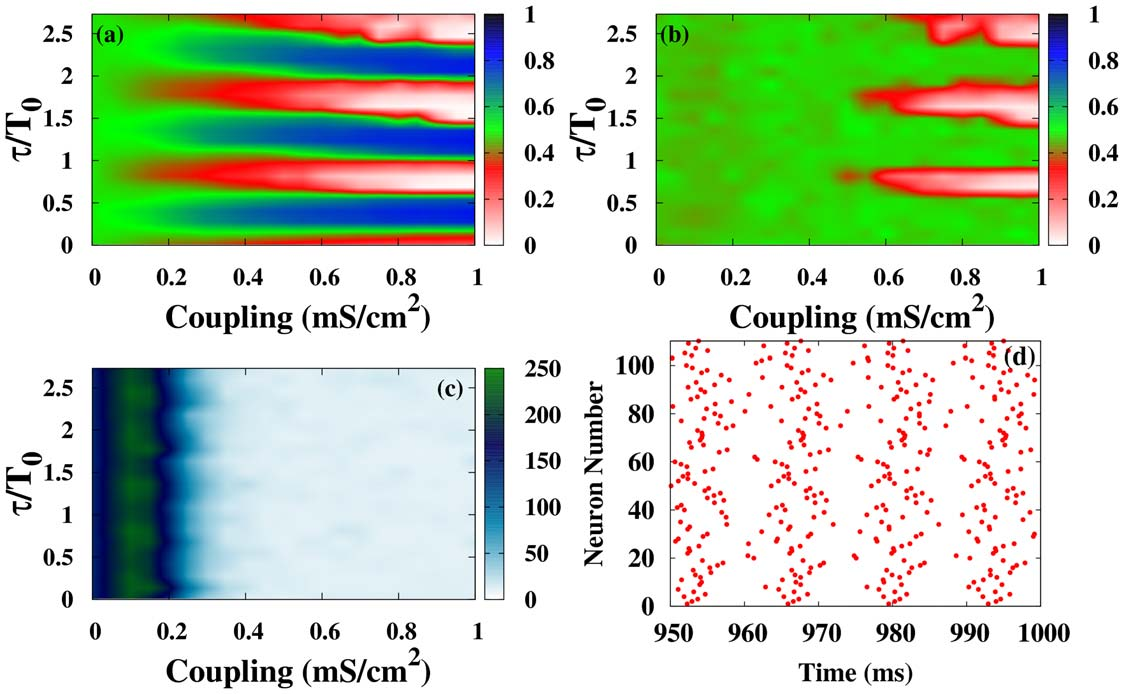
Synchronization regions, number of active neurons and raster plot for a RN with distribution of the frequencies. Contour plot of (a) 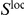 and (b) 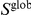 in the coupling strength-delay time phase space for a heterogeneous ensemble of neurons in a RN. (c) Density plot of the number of non-spiking neurons. (d) Raster plot of the activity in the network for a delay time 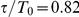 and coupling strength 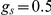 mS cm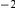.

The assumption that all neurons in the network are identical and operate in a regular spiking regimen is an ideal situation. For this reason, in what follows we explore the case where the natural frequencies of the neurons differ from each other. In the HH model, the frequency of the spikes of the neurons depends on the injection current 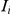. Thus, we model the dispersion in frequencies by assuming that each neuron receives an external current whose value is chosen from a Gaussian distribution with mean 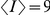 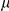 A/cm^2^ and dispersion 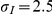 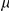 A/cm^2^. With these distribution values 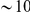
% of the neurons are in the excitable, sub-threshold, state when uncoupled. This distribution of natural frequencies requires an increase of the coupling strength to achieve the synchronous state (see Fig. 7(a-b)). The regions of global synchronization are reduced for most of the networks except in the fully connected one, where the synchronization regions quasi merge at high coupling values, losing the resonant character of the delay. As in the case of identical frequencies, we observe the three different local firing states: in-phase, out-of phase and anti-phase. At a global level, in-phase synchronization is more difficult to achieve and only the random and scale-free networks exhibit this state at high coupling strength for some particular values of the delay time. In all the cases, the predominant state is the one in which the neurons spike out-of-phase, indicated by green areas in Fig. 7(a–b). Another peculiarity that occurs due to the distribution of frequencies is that some neurons become silent, *i.e.*, change from a spiking oscillatory regime to a stable subthreshold state as the interaction strength increases. As we mentioned before, without interaction 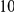
% of the neurons are in a resting state (see Fig. 7(c)). When the coupling strength is increased, the number of silent neurons increases reaching a maximum value for a synaptic strength of 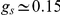 mS cm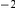. By increasing further the coupling, all neurons reach the regular firing regime again although the raster plot in Fig. 7(d) reveals a high dispersion in their firing phases. Experimental and theoretical investigations have reported the suppression of repetitive firing by short pulses in single neurons [37], [38]: a stable fixed point coexists with two periodic solutions (one stable and one unstable) for a particular range of the injected current in the HH model [38]. Our results suggest that this effect can also be present in other recurrent neuronal networks.

### Anatomical network case

**Figure 8:**
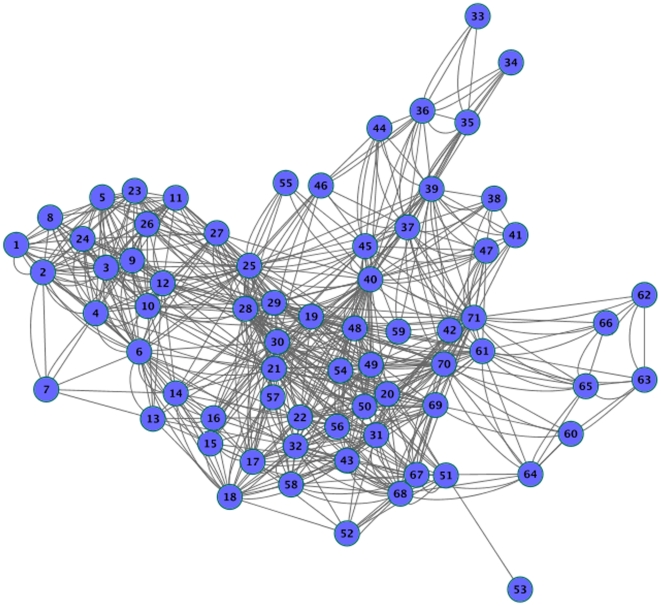
Representation of the macaque cortico-cortical network. Macaque cortical connectivity in an organic layout view.

As an example of real anatomical network we investigate the synchronization in the macaque cortico-cortical network [39]. The network is composed by 71 nodes representing different cortical areas with 746 links between them. Figure 8 shows an organic layout of the cortical connectivity data set.

**Figure 9:**
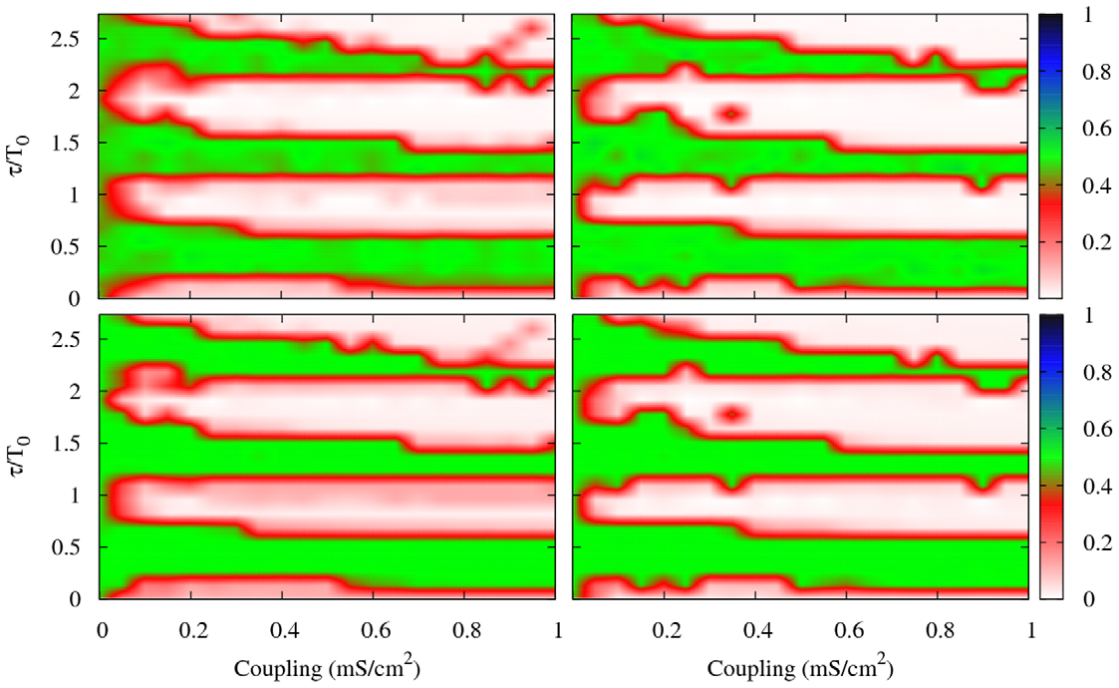
Syncronization regions for the macaque cortical network and its randomized network. Contour plots of 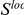 (top panel) and 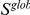 (bottom panel) in the coupling-delay phase space for the macaque anatomical network. Left column: original macaque 71 cortico-cortical network. Right column: randomized version of macaque 71 cortico-cortical network preserving in and out node degree.

We consider each node of the network following the HH neuronal dynamic as described in previous sections. We estimate the degree of synchronization in the network using Eqs. (9) and Eq. (11). Figure 9 shows 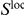 and 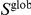 when the coupling between the neurons and the delay time of the connections are varied. We compare the results with a randomized version of the network preserving the degree distribution.

It is interesting to notice that 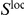 and 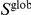 are practically identical. A reason for this is that the macaque cortical network is densely connected, about 15% of the possible links. This result is in agreement with that obtained in previous section, where the global synchronization converges to the local one for a percentage of connected neurons larger than 10% (see Fig. 5). The second remarkable feature is the coincidence of the synchronization regions between the macaque network and the randomized version of the network. The macaque cortical network is very dense and has already an average path length very similar to that of the randomized network (see Table 1) what makes not surprising this result. Another interesting feature is the absence of anti-phase states. This is in accordance with previous observations for densely connected networks, having the extreme limit in the all-to-all network, for which the absence of anti-phase is observed. Densely connected networks present predominantly in-phase synchronization and the resonant role of the delay tends to be less pronounced. Based only on the macaque network we cannot conclude if these facts are general features of live brains, but it raises the interesting question whether this happens in other anatomical networks.

**Table 1:** Macaque cortical and randomized network properties.

Metric	Clustering coefficient	Average degree	Average shortest path
Macaque Network	0.46	10.5	2.33
Randomized version	0.24	10.5	2.06

## Discussion

We have performed numerical simulations of delay-coupled neurons described by the HH and CS models. We have initially assumed that the neurons were chemically coupled and embedded in different complex networks. Our results show that, at a local level, all the considered topologies exhibit three different dynamical regimes: in-phase, anti-phase and out-of-phase. At a global level, however, only networks with certain degree of randomness in the connectivity (in particular random and scale-free networks) allow for a coherent response. These results were also observed when considering electrical coupling, highlighting the generality of the network synchronizing mechanisms. Besides the network architecture, axonal conduction delays also play an important role in the generation of coherent dynamics. We found that such communication latencies do not simply add to the phase difference obtained in the non-delayed case but can determine whether the interconnected cells can set in a coherent firing at all. We expect that our results provide insights in more complex situations, for instance in the presence of distributed delays.

When neurons are not identical, but their natural frequencies are distributed, the region of phase synchronization decreases. Moreover, we found that the number of active neurons decreases for low coupling strengths due to the different frequencies. This emphasizes the importance of having small diversity in the system to obtain a coherent response. Besides the mechanisms studied here, other aspects could be considered as well. Inhibitory neurons and heterogeneous delays might play a significant role and will be considered in detail in future studies.

## Supporting Information

Text S1 **The parameter for both models are shown in Table S1 (HH) and Table S2 (CS).**
(TEX)

Table S1 **Parameters of the Hodgkin-Huxley model.**
(PDF)

Table S2 **Parameters of the Connor-Stevens model.**
(PDF)
