# Supplementary material for: Effect of the Topology and Delayed Interactions in Neuronal Networks Synchronization
Source: PLoS One. 2011 May 27;6(5):e19900. doi: 10.1371/journal.pone.0019900 (PMC3103524; doi:10.1371/journal.pone.0019900)
Supplement: Text S1 — The parameter for both models are shown in Table S1 (HH) and Table S2 (CS). (TEX) [file pone.0019900.s001.tex]

Supporting Material

The parameter for both models are shown in Table 1 (HH) and Table 2 (CS).

Table 1

Parameter	Value	Units
C_m	10	nF/mm^2
g_K	0.36	mS/mm^2
g_{Na}	1.2	mS/mm^2
g_L	0.003	mS/mm^2
V_K	-77	mV
V_{Na}	50	mV
V_L	-54.387	mV

Parameters of the Hodgkin-Huxley model.

Table 2

Parameter	Value	Units
C_m	10	nF/mm^2
g_K	0.2	mS/mm^2
g_{Na}	1.2	mS/mm^2
g_L	0.003	mS/mm^2
g_a	0.477	mS/mm^2
V_K	-72	mV
V_{Na}	55	mV
V_L	-17	mV
V_a	-75	mV

Parameters of the Connor-Stevens model.

The experimentally fitted voltage-dependent transition rates for the HH model, in units of 1/ms with V in units of mV, are

$$\alpha_m(V) = \frac{0.1(V+40)}{1-\exp\left(-(V+40)/10\right)} \quad \beta_m(V) = 4\exp\left(-(V+65)/18\right), \tag{1}$$

$$\alpha_h(V) = 0.07\exp\left(-(V+65)/20\right) \quad \beta_h(V) = \left[1+\exp\left(-(V+35)/10\right)\right]^{-1}, \tag{2}$$

$$\alpha_n(V) = \frac{0.01(V+55)}{1-\exp\left(-0.1(V+55)\right)} \quad \beta_n(V) = 0.125\exp\left(-(V+65)/80\right). \tag{3}$$

These functions, for the CS model are

$$\alpha_m(V) = \frac{0.38(V+29.7)}{1-\exp(-0.1(V+29.7))} \quad \beta_m(V) = 15.2\mathrm{exp}(-0.0556(V+54.7)), \tag{4}$$

$$\alpha_h(V) = 0.266\mathrm{exp}(-0.05(V+48)) \quad \beta_m(V) = 3.8/(1+\exp(-0.1(V+18))), \tag{5}$$

$$\alpha_n(V) = \frac{0.02(V+45.7)}{1-\exp(-0.1(V+45.7))} \quad \beta_m(V) = 0.25\mathrm{exp}(-0.0125(V+55.7)). \tag{6}$$

The A-current is described in terms of the asymptotic values and τ_z functions for its gating variables, with τ_a and τ_b in units of ms and V in units of mV,

$$a_\infty = \left(\frac{0.0761\exp(0.0314(V+94.22))}{1-\exp(0.0346(V+1.17))}\right)^{1/3}, \tag{7}$$

$$\tau_a = 70.3632 + 1.158/(1+\exp(0.0497(V+55.96))), \tag{8}$$

$$b_\infty = \left(\frac{0.0761\exp(0.0314(V+94.22))}{1-\exp(0.0346(V+1.17))}\right)^{1/3}, \tag{9}$$

$$\tau_b = 1.24 + 2.678/(1+\exp(0.0624(V+50))). \tag{10}$$
