# Supplementary material for: Effect of the Topology and Delayed Interactions in Neuronal Networks Synchronization
Source: PLoS One. 2011 May 27;6(5):e19900. doi: 10.1371/journal.pone.0019900 (PMC3103524; doi:10.1371/journal.pone.0019900)
Supplement: Table S2 — Parameters of the Connor-Stevens model. (PDF) [file pone.0019900.s003.pdf]

Supporting Material

Table 1

Parameter	Value	Units
C_m	10	nF/mm^2
g_K	0.2	mS/mm^2
g_{Na}	1.2	mS/mm^2
g_L	0.003	mS/mm^2
g_a	0.477	mS/mm^2
V_K	-72	mV
V_{Na}	55	mV
V_L	-17	mV
V_a	-75	mV

Parameters of the Connor-Stevens model.
